# Post-hypoglycemic nocturnal hyperglycemia in type 1 diabetes: the Somogyi hypothesis revisited

**DOI:** 10.1007/s42000-025-00680-0

**Published:** 2025-06-04

**Authors:** Tomás González-Vidal, Diego Rivas-Otero, Pablo Agüeria-Cabal, Guillermo Ramos-Ruiz, Carmen Lambert, Jessica Ares-Blanco, Edelmiro Menéndez-Torre, Elías Delgado

**Affiliations:** 1 Department of Endocrinology and Nutrition, Hospital Universitario Central de Asturias/University of Oviedo, Oviedo, Spain; 2 Instituto de Investigación Sanitaria del Principado de Asturias (ISPA), Oviedo, Spain; 3 Department of Medicine, University of Oviedo, Oviedo, Spain; 4 Centre for Biomedical Network Research On Rare Diseases (CIBERER), Instituto de Salud Carlos III, Madrid, Spain

**Keywords:** Nocturnal hypoglycemia, Type 1 diabetes, Continuous glucose monitoring, Somogyi

## Abstract

**Purpose:**

The plausibility of the Somogyi phenomenon (dawn hyperglycemia after nocturnal hypoglycemia) has been questioned. The present study used continuous glucose monitoring in patients with type 1 diabetes (T1DM) to investigate the frequency and the associated factors for post-hypoglycemic nocturnal hyperglycemia (PHNH), as well as the overall glycemic control in patients who develop PHNH.

**Methods:**

This study analyzed the nighttime (0:00 am to 6:00 am) glycemic profile of 755 *FreeStyle Libre 2* users with T1DM (429 men; median age 49 years, range 18–90 years) during a 14-day period. Patients were divided into three categories, as follows: no nocturnal hypoglycemia (< 70 mg/dL), only nocturnal hypoglycemia that was not followed by hyperglycemia (> 180 mg/dL) before 6:00 am, and ≥ 1 episode of nocturnal hypoglycemia that was followed by hyperglycemia before 6:00 am (PHNH). The patients' characteristics and the overall glycemic control in the 14-day period were also registered.

**Results:**

A total of 248 patients (32.8%) developed PHNH during the 14-day period. Compared with patients who only had nocturnal hypoglycemia that was not followed by hyperglycemia (n = 332), patients with PHNH were younger, were less frequently diagnosed as latent autoimmune diabetes in adults (LADA), and used higher total daily doses of insulin. Patients with PHNH had longer time above range, shorter time in range, higher glucose variability, and more diurnal hypoglycemia than those who only had nocturnal hypoglycemia that was not followed by hyperglycemia before 6:00 am.

**Conclusions:**

PHNH is frequent in T1DM, especially in young individuals. Compared to patients with other forms of nocturnal hypoglycemia, patients with PHNH have poorer glycemic control.

## Introduction

Hypoglycemia can cause unpleasant symptoms that can progress to loss of consciousness, seizures, coma, or even death [1]. In addition, hypoglycemia is known to be associated with long-term adverse outcomes in people with diabetes, including increased mortality in different settings [2–5]. For these reasons, hypoglycemia can lead to anxiety and fear in people with diabetes [6, 7]. Nearly 50% of the hypoglycemic episodes occur at night while the person with diabetes is asleep [8], making nocturnal hypoglycemia a particular source of concern [9, 10].

The Somogyi effect, a hypothesis proposed by Michael Somogyi in 1938 [11, 12], suggests that insulin-induced nocturnal hypoglycemia might stimulate the secretion of counterregulatory hormones [13, 14] or induce eating [15], resulting in rebound nocturnal hyperglycemia that the patient with diabetes would often detect at dawn, upon awakening [12]. This hypothesis of post-hypoglycemic nocturnal hyperglycemia (PHNH) has since then been questioned [15–21]. However, experimental studies have validated this hypothesis [22], which has been widely accepted for decades [23].

To our knowledge, no studies have analyzed the frequency and associated factors of PHNH or the glycemic control of patients who develop PHNH. PHNH is a specific form of nocturnal hypoglycemia that may have its own associated factors, which might not be the same as those associated with any form of nocturnal hypoglycemia [24–27]. The present study used continuous glucose monitoring (CGM) to determine the prevalence and associated factors of PHNH in type 1 diabetes mellitus (T1DM) as well as the overall glycemic control of patients with T1DM who develop PHNH.

## Materials and methods

### Study design and setting

From a sample of 873 *FreeStyle Libre 2* (FSL2, Abbott, USA) users with T1DM treated in the endocrinology department of a university hospital in Spain, described elsewhere [28–30], a total of 755 patients (429 men [56.8%]; median age 49 years, range 18–90 years) with sensor usage ≥ 70% [31] were selected for this cross-sectional study. T1DM was defined by the presence of a diagnosis of diabetes [32], evidence of pancreatic islet autoimmunity (positivity for autoantibodies to the glutamic acid decarboxylase, the tyrosine phosphatase-related islet antigen 2, or the zinc transporter 8) [32], and insulin requirement, given that only patients with insulin dependency were included. Therefore, subjects with stage 1 of T1DM (evidence of pancreatic autoimmunity but normoglycemia, thus no insulin requirement) [32] were excluded. Patients younger than 18 years and pregnant women were also excluded.

*FreeStyle Libre 2* is an intermittently scanned CGM system commercialized by Abbott in 2018 [33]. The system provides interstitial glucose measurements via a 14-day lifetime sensor [33]. In the Spanish region of Asturias, where this study was conducted, FSL2 devices are financed by the public healthcare system for people with T1DM. Data were recorded through *LibreView*, a cloud-based platform that enables FSL2 users to share their glucose data with healthcare practitioners. Data were collected from August 18 to September 21, 2023, selecting the available 14-day period closest to the data collection dates, with a maximum of 1 year prior the data collection date (the oldest piece of data was from August 27, 2022). For patients with sensor usage so low that the system was unable to calculate the glucose management indicator (GMI), the available 14-day period closest to the data collection date that permitted GMI calculation was selected.

### Main determinations

**Fig. 1:**
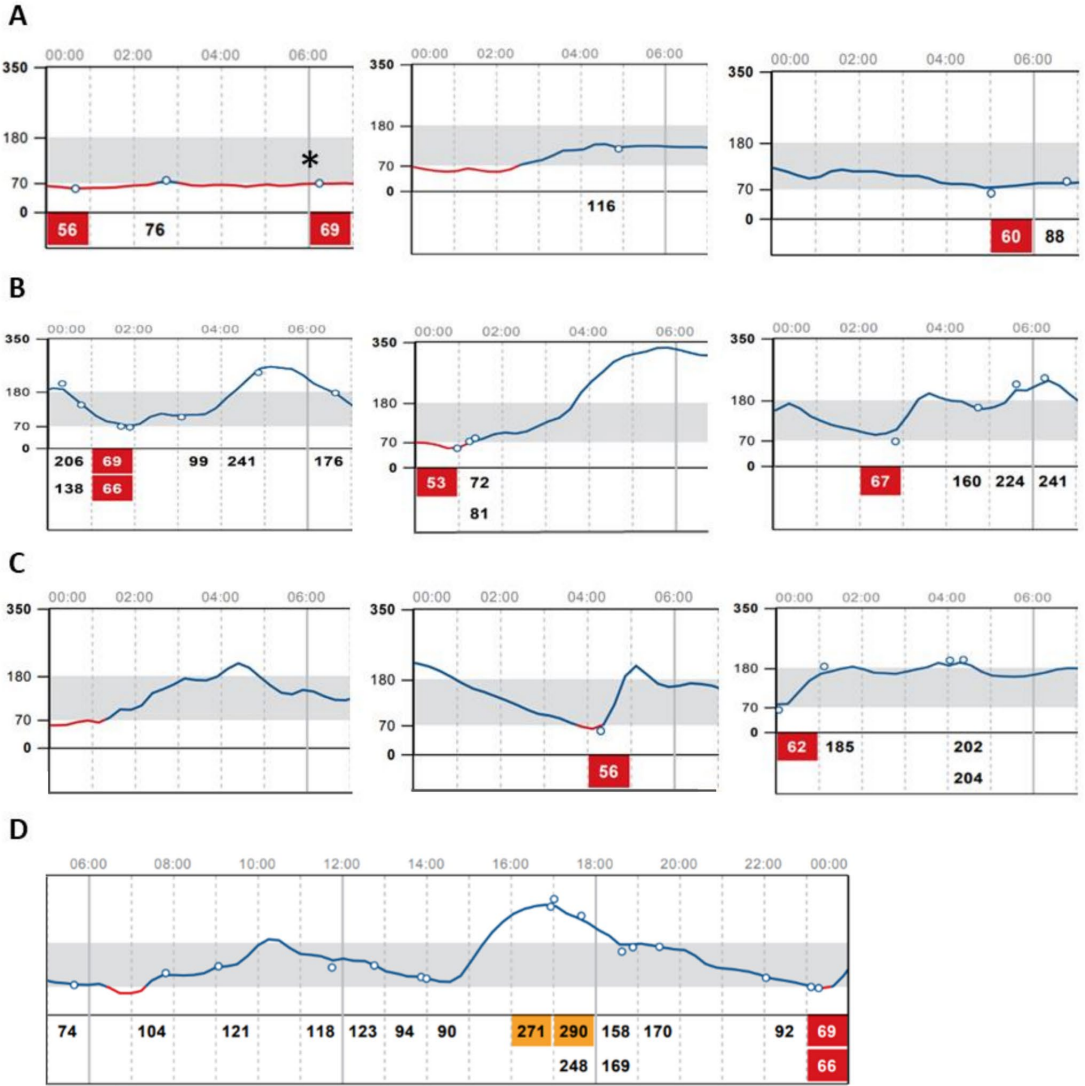
Examples of how diurnal and nocturnal hypoglycemia were defined. **(A)** Nocturnal hypoglycemia that was not followed by hyperglycemia before 6:00 am. Episodes like the one marked with an asterisk, where nocturnal hypoglycemia extended beyond 6:00 am, were classified as both nocturnal and diurnal hypoglycemia. **(B)** PHNH with hyperglycemia at 6:00 am. **(C)** PHNH without hyperglycemia at 6:00 am. **(D)** Diurnal (6:01 am to 23:59 pm) hypoglycemia

Patients were divided into three categories according to the presence of different forms of nocturnal hypoglycemia during the 14-day period, as follows: no nocturnal hypoglycemia, only nocturnal hypoglycemia that was not followed by hyperglycemia before 6:00 am, and ≥ 1 episode of nocturnal hypoglycemia that was followed by hyperglycemia before 6:00 am (PHNH). The number of nights (0:00 am to 6:00 am) with nocturnal hypoglycemic episodes during the 14-day period was recorded, differentiating hypoglycemia that was not followed by hyperglycemia before 6:00 am and PHNH. A patient was considered to have had a night with an episode of nocturnal hypoglycemia when the *LibreView* graph recorded a glucose value of < 70 mg/dL (either when a glucose determination of < 70 mg/dL was recorded, or when the glucose line went below the 70 mg/dL limit) between 0:00 am and 6:00 am, regardless of its duration (Fig. 1, panels A, B and C). Episodes of nocturnal hypoglycemia were considered PHNH when nocturnal hypoglycemia (hypoglycemia between 0:00 am and 6:00 am) was followed by a glucose value of > 180 mg/dL (either when a glucose determination of > 180 mg/dL was recorded or when the glucose line went above the > 180 mg/dL limit) before 6:00 am (Fig. 1, panels B and C). PHNH episodes were further differentiated by whether hyperglycemia was maintained at 6:00 am or whether hyperglycemia was corrected before 6:00 am (Fig. 1, panels B and C).

### Covariates

*CGM-related variables.* Percentages of the day during which the patient had certain interstitial glucose concentrations (time above range level 2 [> 250 mg/dL], time above range level 1 [181–250 mg/dL], time in range [70–180 mg/dL], time below range level 1 [54–69 mg/dL], and time below range level 2 [< 54 mg/dL]), GMI (this indicates the patient's estimated glycated hemoglobin level based on the mean glucose level), glucose variability (GV, expressed as the coefficient of variation) [31], number of hypoglycemic events (glucose levels < 70 mg/dL during at least 15 min) that occurred during the 14-day period and its mean duration, number of days during the 14-day period with diurnal hypoglycemic episodes (as recorded in the *LibreView* graph from 6:01 am to 23:59 pm; Fig. 1, panel D), scans per day (number of times per day the patient scanned the FSL2 device to check the glucose levels), and use of hypoglycemia alarms of the FSL2 devices (whether the alarm was set and, if so, the threshold [mg/dL] at which it was set).

*Patient characteristics.* Age, sex, duration of diabetes (calculated in years from the time of diagnosis of diabetes according to the American Diabetes Association criteria) [32], diagnosis of latent autoimmune diabetes in adults (LADA, a subtype of antibody-positive T1DM defined as adult-onset diabetes [> 30 years of age at diagnosis] with no need for insulin treatment for at least 6 months after diagnosis) [34], presence of advanced chronic complications of diabetes (including microvascular complications such as diabetic retinopathy [proliferative diabetic retinopathy, diabetic retinopathy with macular edema, and diabetic retinopathy that required photocoagulation], diabetic kidney disease that required evaluation by a nephrologist, and diabetic peripheral neuropathy; and macrovascular complications such as peripheral artery disease that required evaluation by a vascular surgeon, cerebrovascular disease, myocardial infarction, and heart failure), body mass index, and smoking status.

*Insulin therapy*. The total daily dose of insulin (TDDI), expressed in international units, was recorded. A total of 32 (4.2%) patients were treated with an insulin pump (open-loop insulin pumps only, given that closed-loop systems cannot be integrated with the FSL2 system) [35] and 723 (95.8%) received subcutaneous insulin. Among patients on subcutaneous insulin, 695 (96.1%) used basal-bolus therapy, 21 (2.9%) used basal insulin alone, six (0.8%) used premixed insulins, and one (< 0.1%) used only rapid-acting insulin. For patients on basal-bolus therapy, the specific types of basal and rapid-acting insulins were recorded, distinguishing next-generation basal insulins (degludec and U-300 glargine) from earlier-generation formulations (detemir and U-100 glargine), and next-generation rapid-acting insulins (faster-aspart) from earlier-generation formulations (aspart, glulisine, lispro, and human insulin). Additionally, the timing of basal insulin administration was recorded for patients on basal-bolus therapy.

### Statistical analyses

We employed the chi-square test to compare proportions (with Yates’ correction applied for 2 × 2 contingency tables when the expected frequency in any cell was less than 5), the Mann–Whitney test to compare numerical data between two independent groups, and Spearman’s rank test to assess correlation. Logistic regression was used for multivariate analyses of factors associated with PHNH. All tests were two-tailed and P-values lower than 0.05 were considered statistically significant. Data were analyzed using IBM SPSS Statistics version 20 (IBM Corp., USA).

## Results

### Frequency of PHNH

**Fig. 2:**
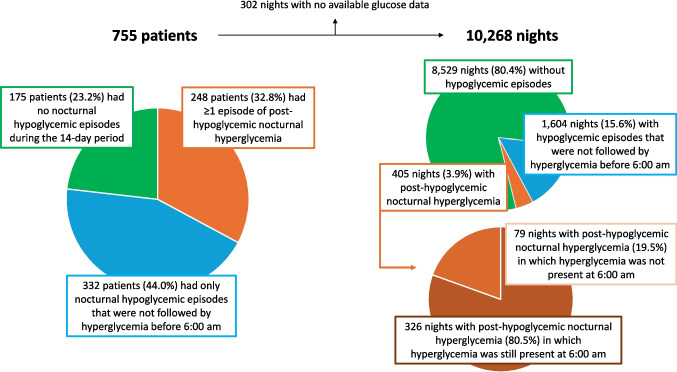
Frequency of different forms of nocturnal hypoglycemia during the 14-day periods

A total of 580 patients (76.8%) had any form of nocturnal hypoglycemic episodes during the 14-day period: 248 (32.8%) developed ≥ 1 episode of PHNH and 332 (44.0%) developed only episodes of nocturnal hypoglycemia that were not followed by hyperglycemia before 6:00 am (Fig. 2, left panel). A total of 10,268 nights of the 755 patients were evaluated during the 14-day periods given that 302 nights had no available glucose data. Hypoglycemic episodes occurred in 2,009 nights (19.5%): PHNH episodes occurred in 405 nights (3.9%) and hypoglycemic episodes that were not followed by hyperglycemia before 6:00 am occurred in 1,604 nights (15.6%) (Fig. 2, upper right panel). Among the 405 nights with PHNH, in 326 nights (80.5%) hyperglycemia was still present at 6:00 am (Fig. 2, bottom right panel).

Among patients who experienced ≥ 1 episode of any form of nocturnal hypoglycemia (n = 580), the mean number of episodes of any form of nocturnal hypoglycemia during the 14-day period was 3.4 episodes (standard deviation 2.3 episodes; range 1–12 episodes). Among patients who experienced ≥ 1 episode of PHNH (n = 248), the mean number of episodes of PHNH in the 14-day period was 1.6 episodes (standard deviation 0.9 episodes; range 1–6 episodes).

### Factors associated with PHNH

Table 1 displays a comparison of the characteristics of patients who had ≥ 1 episode of any form of nocturnal hypoglycemia and patients who did not have nocturnal hypoglycemia. Patients who had ≥ 1 episode of nocturnal hypoglycemia were younger, were less frequently diagnosed as LADA, and set hypoglycemia alarms at lower thresholds (Table 1). Patients with nocturnal hypoglycemia also tended to have a lower use of hypoglycemia alarms, to be treated with open-loop insulin pumps (rather than subcutaneous insulin therapy), and, among those on basal-bolus therapy, to be treated with a lower proportion of basal insulin (i.e., a higher proportion of rapid-acting boluses) [29].

**Table 1:** Patients’ characteristics and glycemic control according to the presence of episodes of any form of nocturnal hypoglycemia

**Variable**	**No nocturnal hypoglycemia** (n = 175)	** ≥ 1 episode of any form of nocturnal hypoglycemia** (n = 580)	**P value**
**Demographic variables**			
**Sex** (man)	91 (52.0)	338 (58.3)	0.142
**Age** (years)	53 (16)	47 (14)	< 0.001
**Metabolic and lifestyle variables**			
**Duration of diabetes** (years)	20 (13)	22 (13)	0.070
**LADA** (yes)^a^	45 (25.7)	69 (11.9)	< 0.001
**Body mass index** (kg/m^2^)^b^	26.7 (4.9)	26.8 (4.7)	0.885
**Ever smoker** (yes)^c^	75 (44.4)	241 (42.9)	0.731
**Current smoker** (yes)^c^	32 (18.9)	123 (21.9)	0.411
**Advanced chronic complications of diabetes**			
**Microvascular complications** (yes) **Diabetic retinopathy** (yes) **Diabetic kidney disease** (yes) **Diabetic peripheral neuropathy** (yes)	33 (18.9) 28 (16.9) 11 (6.3) 9 (5.1)	95 (16.4) 83 (14.3) 31 (5.3) 25 (4.3)	0.444 0.580 0.634 0.642
**Macrovascular complications** (yes) **Myocardial infarction** (yes) **Heart failure** (yes) **Cerebrovascular disease** (yes) **Peripheral artery disease** (yes)	22 (12.6) 10 (5.7) 6 (3.4) 7 (4.0) 11 (6.3)	54 (9.3) 22 (3.8) 15 (2.6) 16 (2.8) 29 (5.0)	0.209 0.269 0.553 0.402 0.506
**Insulin treatment**			
**Total daily dose of insulin** (IU/kg/day)^d^	0.67 (0.27)	0.66 (0.26)	0.495
**Basal insulin alone** (yes)	10 (5.7)	11 (1.9)	0.015
**Percentage of basal insulin for patients on basal-bolus therapy** (%)^e^	55.8 (12.1)	53.4 (13.4)	0.061
**Next-generation basal insulin for patients on basal-bolus therapy** (yes)^e^	147 (91.9)	490 (91.6)	0.909
**Next-generation rapid-acting insulin for patients on basal-bolus therapy** (yes)^e^	50 (31.2)	171 (32.0)	0.895
**Open-loop insulin pump** (yes)	3 (1.7)	29 (5.0)	0.059
**Continuous glucose monitoring use**			
**Hypoglycemia alarm set** (yes)	138 (78.9)	419 (72.2)	0.081
**Hypoglycemia alarm threshold** (mg/dL)^f^	78.0 (8.7)	73.3 (6.9)	< 0.001
**Scans per day** (n)	10.9 (8.6)	10.5 (6.2)	0.743
**Glycemic control (continuous glucose monitoring)**			
**Time above range, level 2** (> 250 mg/dL) (%)	19.2 (17.4)	11.7 (10.8)	< 0.001
**Time above range, level 1** (181–250 mg/dL) (%)	28.7 (9.9)	23.1 (8.2)	< 0.001
**Time in range** (70–180 mg/dL) (%)	51.3 (21.5)	60.3 (15.5)	< 0.001
**Time below range, level 1** (54–69 mg/dL) (%)	0.6 (1.0)	4.0 (3.4)	< 0.001
**Time below range, level 2** (< 54 mg/dL) (%)	0.0 (0.2)	0.6 (1.4)	< 0.001
**Glucose management indicator** (%)	7.8 (0.9)	7.2 (0.6)	< 0.001
**Glucose variability** (%)	31.9 (5.8)	38.1 (6.1)	< 0.001
**Hypoglycemic events** (n)^g^	1.9 (2.7)	8.9 (6.5)	< 0.001
**Duration of hypoglycemia** (min)^h^	66 (38)	100 (49)	< 0.001
**Days with diurnal hypoglycemic episodes** (n)^i^	3.0 (2.7)	7.2 (3.3)	< 0.001

Data are expressed as mean and standard deviation (within parentheses) or as absolute numbers and percentage (within parentheses). P-values were obtained using the Mann–Whitney test (for numerical variables) and the chi-square test (for categorical variables; with Yates’ correction applied when the expected frequency in any cell was < 5)

^a^Subtype of antibody-positive type 1 diabetes that is defined as adult-onset diabetes (> 30 years at diagnosis) with absence of insulin requirement for at least 6 months after diagnosis

^b^Data available for 752 patients

^c^Data available for 731 patients

^d^Data available for 735 patients

^e^Data available for 695 patients given that only patients on basal-bolus therapy were selected for these analyses. The percentage of basal insulin was calculated as daily dose of basal insulin/(daily dose of basal insulin + daily dose of bolus insulin)

^f^Data available for 557 patients given that only patients with the hypoglycemia alarm set were selected

^g^Number of hypoglycemic episodes with a duration of more than 15 min during the 14-day period

^h^Data available for 671 patients as only patients with at least 1 hypoglycemic event of more than 15 min of duration were selected (duration of hypoglycemia is considered 0 min in those with no hypoglycemic events)

^i^Number of days (6:01 am to 23:59 pm period) during the 14-day period in which the *LibreView* graph recorded at least 1 hypoglycemic episode, regardless of its duration

IU, international units. LADA, latent autoimmune diabetes in adults

Table 2 includes only patients who had ≥ 1 episode of any form of nocturnal hypoglycemia and compares the characteristics of patients who had ≥ 1 episode of PHNH with those who only had hypoglycemia that was not followed by hyperglycemia before 6:00 am. Patients who had ≥ 1 episode of PHNH were younger, were less frequently diagnosed as LADA, and used higher TDDI (Table 2). However, in multivariate analyses (logistic regressions) performed in the entire study population, only age and LADA diagnosis maintained a negative independent association with PHNH, while the TDDI did not (Table 3). Similarly, other characteristics of the insulin regimens were not associated with the frequency of PHNH (Table 2). Additionally, the timing of basal insulin administration in patients on basal-bolus therapy was not associated with nocturnal hypoglycemia or PHNH, regardless of whether next- or earlier-generation formulations were used (data not shown). Among the six patients treated with premixed insulins, two experienced nocturnal hypoglycemia, but none developed PHNH. Diabetic microvascular complications, particularly retinopathy, were also associated with PHNH (Table 2).

**Table 2:** Patients’ characteristics and glycemic control among patients with episodes of nocturnal hypoglycemia, according to the presence of PHNH

	**Patients with episodes of nocturnal hypoglycemia** (n = 580)		
**Variable**	**No PHNH** (n = 332)	** ≥ 1 episode of PHNH** (n = 248)	**P value**
**Demographic variables**			
**Sex** (man)	190 (57.2)	148 (59.7)	0.554
**Age** (years)	49 (15)	46 (13)	0.019
**Metabolic and lifestyle variables**			
**Duration of diabetes** (years)	22 (13)	22 (12)	0.601
**LADA** (yes)^a^	48 (14.5)	21 (8.5)	0.027
**Body mass index** (kg/m^2^)^b^	26.9 (4.8)	26.6 (4.7)	0.375
**Ever smoker** (yes)^c^	142 (43.7)	99 (41.8)	0.650
**Current smoker** (yes)^c^	65 (20.0)	58 (24.5)	0.205
**Advanced chronic complications of diabetes**			
**Microvascular complications** (yes) **Diabetic retinopathy** (yes) **Diabetic kidney disease** (yes) **Diabetic peripheral neuropathy** (yes)	45 (13.6) 39 (11.7) 19 (5.7) 12 (3.6)	50 (20.2) 44 (17.7) 12 (4.8) 13 (5.2)	0.033 0.041 0.640 0.340
**Macrovascular complications** (yes) **Myocardial infarction** (yes) **Heart failure** (yes) **Cerebrovascular disease** (yes) **Peripheral artery disease** (yes)	27 (8.1) 11 (3.3) 10 (3.0) 10 (3.0) 13 (3.9)	27 (10.9) 11 (4.4) 5 (2.0) 6 (2.4) 16 (6.5)	0.259 0.484 0.455 0.666 0.166
**Insulin treatment**			
**Total daily dose of insulin** (IU/kg/day)^d^	0.65 (0.29)	0.67 (0.22)	0.022
**Basal insulin alone** (yes)	9 (2.7)	2 (0.8)	0.175
**Percentage of basal insulin for patients on basal-bolus therapy** (%)^e^	53.0 (13.6)	53.9 (13.2)	0.534
**Next-generation basal insulin for patients on basal-bolus therapy** (yes)^e^	273 (90.7)	217 (92.7)	0.400
**Next-generation rapid-acting insulin for patients on basal-bolus therapy** (yes)^e^	90 (29.9)	81 (34.6)	0.246
**Open-loop insulin pump** (yes)	17 (5.1)	12 (4.8)	0.878
**Continuous glucose monitoring use**			
**Hypoglycemia alarm set** (yes)	235 (70.8)	184 (74.2)	0.364
**Hypoglycemia alarm threshold** (mg/dL)^f^	73.5 (7.1)	73.1 (6.6)	0.625
**Scans per day** (n)	10.2 (6.0)	10.9 (6.4)	0.115
**Glycemic control (continuous glucose monitoring)**			
**Time above range, level 2** (> 250 mg/dL) (%)	10.3 (10.3)	13.6 (11.2)	< 0.001
**Time above range, level 1** (181–250 mg/dL) (%)	22.3 (9.0)	24.2 (6.8)	0.033
**Time in range** (70–180 mg/dL) (%)	62.7 (16.2)	57.1 (13.8)	< 0.001
**Time below range, level 1** (54–69 mg/dL) (%)	3.9 (3.5)	4.1 (3.3)	0.245
**Time below range, level 2** (< 54 mg/dL) (%)	0.5 (1.2)	0.8 (1.6)	0.013
**Glucose management indicator** (%)	7.1 (0.6)	7.3 (0.6)	< 0.001
**Glucose variability** (%)	37.1 (5.9)	39.4 (6.2)	< 0.001
**Hypoglycemic events** (n)^g^	8.2 (6.4)	9.8 (6.5)	0.001
**Duration of hypoglycemia** (min)^h^	107 (56)	92 (37)	0.003
**Days with nocturnal hypoglycemic episodes** (n)^i^	3.0 (2.2)	3.9 (2.2)	< 0.001
**Days with diurnal hypoglycemic episodes** (n)^j^	6.9 (3.3)	7.7 (3.2)	0.007

Data are expressed as mean and standard deviation (within parentheses) or as absolute numbers and percentage (within parentheses). P-values were obtained using the Mann–Whitney test (for numerical variables) and the chi-square test (for categorical variables; with Yates’ correction applied when the expected frequency in any cell was < 5)

^a^Subtype of antibody-positive type 1 diabetes that is defined as adult-onset diabetes (> 30 years at diagnosis) with absence of insulin requirement for at least 6 months after diagnosis

^b^Data available for 577 patients

^c^Data available for 562 patients

^d^Data available for 561 patients

^e^Data available for 535 patients given that only patients on basal-bolus therapy were selected for these analyses. The percentage of basal insulin was calculated as daily dose of basal insulin/(daily dose of basal insulin + daily dose of bolus insulin)

^f^Data available for 419 patients given that only patients with the hypoglycemia alarm set were selected

^g^Number of hypoglycemic episodes with a duration of more than 15 min during the 14-day period

^h^Data available for 561 patients as only patients with at least 1 hypoglycemic event of more than 15 min of duration were selected (duration of hypoglycemia is considered 0 min in those with no hypoglycemic events)

^i^Number of nights (0:00 am to 6:00 am period) during the 14-day period in which the *LibreView* graph recorded at least 1 hypoglycemic episode, regardless of its duration

^j^Number of days (6:01 am to 23:59 pm period) during the 14-day period in which the *LibreView* graph recorded at least 1 hypoglycemic episode, regardless of its duration

PHNH, post-hypoglycemic nocturnal hyperglycemia. IU, international units. LADA, latent autoimmune diabetes in adults

**Table 3:** Multivariate analyses (logistic regressions) of factors associated with ≥ 1 episode of PHNH during the 14-day period

**Characteristic**	**Crude analyses**			**Adjusted for age and sex**			**Multiadjusted**^a^		
	**OR**	**95% CI**	**P-value**	**OR**	**95% CI**	**P-value**	**OR**	**95% CI**	**P-value**
**Sex** (man)	1.19	0.87–1.62	0.268	1.14	0.84–1.56	0.387	1.17	0.85–1.62	0.330
**Age** (years)	0.98	0.97–0.99	< 0.001	0.98	0.97–0.99	< 0.001	0.98	0.97–0.99	0.017
**LADA** (yes)^b^	0.41	0.25–0.67	< 0.001	0.54	0.31–0.92	0.026	0.48	0.27–0.84	0.011
**Total daily dose of insulin** (IU/kg/day)	1.33	0.75–2.35	0.325	1.22	0.68–2.18	0.496	1.20	0.67–2.17	0.529

The multivariate logistic regression models included 755 cases, except those including the total daily dose of insulin, which included 735 cases

^a^Adjusted for sex, age, LADA diagnosis, and total daily dose of insulin

^b^Subtype of antibody-positive type 1 diabetes that is defined as adult-onset diabetes (> 30 years at diagnosis) with absence of insulin requirement for at least 6 months after diagnosis

OR, odds ratio. CI, confidence interval. IU, international units. LADA, latent autoimmune diabetes in adults

### PHNH and glycemic control

Table 1 also shows a comparison of the glycemic control of patients who had ≥ 1 episode of any form of nocturnal hypoglycemia and patients who did not have nocturnal hypoglycemia. Patients who had ≥ 1 episode of nocturnal hypoglycemia had a higher GV and, as expected, tighter glycemic control: fewer hyperglycemia features, longer time in range, more hypoglycemia features, and a lower GMI (Table 1).

Table 2 includes only patients who had ≥ 1 episode of any form of nocturnal hypoglycemia and compares the glycemic control of patients who had ≥ 1 episode of PHNH with those who only had hypoglycemia that was not followed by hyperglycemia before 6:00 am. Patients who had ≥ 1 episode of PHNH had longer time above range, shorter time in range, longer time below range level 2, a higher GMI, a higher GV, more hypoglycemic events, a shorter duration of hypoglycemic events, and more episodes of both diurnal and nocturnal hypoglycemia (Table 2). There was a positive correlation between the percentage of nights with PHNH among nights with any form of nocturnal hypoglycemia (number of nights with PHNH divided by the number of nights with any form nocturnal hypoglycemia) and the time above range level 2 (rho = 0.211, p < 0.001), the time above range level 1 (rho = 0.157, p < 0.001), the GMI (rho = 0.230, p < 0.001), and the GV (rho = 0.113, p = 0.006). There was a negative correlation between the percentage of nights with PHNH among nights with any form of nocturnal hypoglycemia and the time in range (rho = −0.207, p < 0.001) and the duration of hypoglycemia (rho = −0.207, p < 0.001).

## Discussion

Our results show that nocturnal hypoglycemia is a frequent problem in patients with T1DM: approximately three-quarters of patients with T1DM had an episode of nocturnal hypoglycemia during a 14-day period. Patients who did not have episodes of nocturnal hypoglycemia had, on average, loose glycemic control, suggesting that nocturnal hypoglycemia remains a common side effect when trying to achieve tight T1DM control. This well-established inverse relationship between mean glucose levels (i.e., GMI) and nocturnal hypoglycemia risk underscores the challenge of achieving optimal glycemic control without increasing the likelihood of hypoglycemia. PHNH was also common, as almost one-third of patients in our study had at least one episode of PHNH over 14 days. In most PHNH episodes, hyperglycemia persisted at dawn (6:00 am), whereas there were few (79/405) PHNH episodes in which hyperglycemia was corrected during nighttime. PHNH was more frequent in younger individuals and was associated with poorer glycemic control.

Any form of nocturnal hypoglycemia was less frequent in patients with older age, in patients who were diagnosed as LADA, and in patients with hypoglycemia alarms set at high thresholds (i.e., above the hypoglycemic range). Regarding insulin therapy, nocturnal hypoglycemia was less frequent in patients on basal insulin alone (as well as in patients on basal-bolus therapy using higher propotions of basal insulin) [29] and tended to be more common in open-loop insulin pump users (who likely sought tighter glycemic control than those on subcutaneous insulin). Among patients who had nocturnal hypoglycemic episodes, those with PHNH had distinctive characteristics: in comparison with patients who only had hypoglycemia that was not followed by hyperglycemia before 6:00 am, patients with PHNH were younger, were less frequently diagnosed as LADA, and used higher TDDI. The setting of hypoglycemia alarms was similar in patients with PHNH and in patients with nocturnal hypoglycemia with no subsequent hyperglycemia; thus, it could be assumed that the number of awakenings caused by alarms was similar in the two groups. However, young patients, who have better hypoglycemia awareness than older patients [36], may have a greater number of awakenings due to symptomatic hypoglycemia, eating later to correct it and producing the PHNH. In addition, the autonomic nervous system of young patients might respond more intensely to hypoglycemia than that of older patients, causing a greater and earlier release of counterregulatory hormones [37], thus favoring PHNH. LADA is a slowly progressive form of T1DM in which pancreatic islet destruction is not abrupt [34]. This slower depletion of pancreatic insulin reserve [34] can facilitate glycemic control and reduce glycemic excursions, which may explain the lower frequency of PHNH observed. Lastly, patients experiencing PHNH used higher TDDI, as hypothesized by Michael Somogyi, who stated that insulin is a cause of extreme hyperglycemia and glucose instability [11, 38]. In our sample, there was also a positive correlation between the TDDI and the GV (i.e., glucose instability; data not shown). However, the association between the TDDI and PHNH was not significant after adjusting for other covariates. Likewise, no significant associations were found between PHNH and other features of the insulin regimens.

As expected based on the characteristics of the phenomenon, patients who experienced PHNH had a longer time above range, a higher GMI, a shorter duration of hypoglycemia (because the episodes of nocturnal hypoglycemia were probably corrected earlier, limiting their progression and reducing the overall time spent below the hypoglycemic threshold), and a higher GV (because ups and downs in glucose levels lead to an increased GV) [39] than patients who only experienced nocturnal hypoglycemia that was not followed by hyperglycemia before 6:00 am. In addition, patients with PHNH also had shorter time in range, higher time below range level 2, and more episodes of diurnal hypoglycemia than patients who had only nocturnal hypoglycemia that was not followed by hyperglycemia before 6:00 am. The latter is remarkable considering that episodes of nocturnal hypoglycemia that persisted at 6:01 am were classified as both nocturnal hypoglycemia and diurnal hypoglycemia (Fig. 1, panel A). A higher time below range level 2 in patients with PHNH might indicate that overcorrection of hypoglycemia can be more common after pronounced hypoglycemia (< 54 mg/dL), which may lead to either an increased counterregulatory hormonal response or increased food intake. Overall, our results show that PHNH is a form of nocturnal hypoglycemia that is associated with particularly poor and unstable T1DM control. In fact, PHNH was also associated with microvascular complications of diabetes and particularly advanced diabetic retinopathy, in line with existing evidence that a high GV and acute glucose fluctuations contribute to retinal damage [40–42]. This finding is noteworthy given that patients who developed PHNH were younger, despite diabetic complications typically being associated with older age [30, 43].

Our study has some limitations, including those inherent to its retrospective design [44]. Although the CGM data were extracted from a platform that allowed identification of hypoglycemia (< 70 mg/dL) and hyperglycemia (> 180 mg/dL) between 0:00 am and 6:00 am (Fig. 1, panels A, B, and C), it did not provide sufficient temporal detail to retrieve all glucose values with precise time annotations. As a result, interval-based analyses of glycemic control could not be performed, which would have been particularly valuable for characterizing nocturnal glycemic patterns. We could not determine whether hyperglycemia following nocturnal hypoglycemia was due to carbohydrate intake, a counterregulatory hormonal response, or a combination of both mechanisms. We were also unable to assess the impact of physical activity on PHNH, despite its known potential to increase the risk of hypoglycemia in people with T1DM, both during exercise and for several hours afterwards [45, 46]. According to the consensus recommendations for clinical studies with CGM, patients with sensor usage < 70% were excluded [31] given the high rate of incomplete nocturnal data in individuals with lower sensor usage. The frequency of PHNH could be different in patients with lower sensor usage. Nighttime glucose levels < 70 mg/dL defined nocturnal hypoglycemia regardless of duration, although hypoglycemic episodes of < 15 min on CGM may not always be clinically relevant [31]. The same nocturnal rest period (00:00 am to 6:00 am) was considered for all patients, although some patients might have been awake during these times and/or asleep at other times outside the period. None of the six patients treated with premixed insulins developed PHNH. This finding, likely related to the limited number of patients in this subgroup, warrants further exploration in larger studies comparing this regimen with others, including closed-loop insulin pumps. Strengths of the study include a large sample size and having reviewed more than 10,000 nights of patients with T1DM by CGM.

## Conclusions

Our results can have clinical applications. Regardless of the causative mechanism, which is much debated [15, 16], PHNH is common in patients with T1DM, especially in younger individuals. Thus, PHNH should be ruled out in patients on insulin therapy who present with dawn hyperglycemia, as stated by Michael Somogyi in the twentieth century [12]. Patients with T1DM who develop PHNH represent a distinct subgroup characterized by particularly unstable glycemic control. Future studies are needed to determine the most common causal mechanisms of PHNH, which will allow the development of strategies to prevent this phenomenon in patients with T1DM.

## Data Availability

The datasets used and/or analyzed during the current study are not publicly available due to ethical reasons but are available from the corresponding author on reasonable request.
